# Effect of Organotin on Performance of Siloxane Materials for Surface Treatment of Cementitious Materials

**DOI:** 10.3390/ma18071626

**Published:** 2025-04-02

**Authors:** Kaiyue Huang, Ji Zhang, Yue Li, Hui Yang

**Affiliations:** 1 School of Materials Science and Engineering, Zhejiang University, Hangzhou 310058, China; 12126070@zju.edu.cn (K.H.); yanghui@zju.edu.cn (H.Y.); 2 Zhejiang-California International Nanosystems Institute, Zhejiang University, Hangzhou 310058, China; jizhang@zju.edu.cn; 3 Institute of Wenzhou, Zhejiang University, Wenzhou 325000, China

**Keywords:** cementitious material, siloxane, organotin, surface treatment, hardness

## Abstract

Surface treatment is essential for cementitious materials. Siloxane is a material that can potentially be applied in surface treatment, as it possesses the advantages of both inorganic and organic surface treatment materials for cement. Organotin, serving as a neutral catalyst, performs well in improving the performance of siloxane coating. In this work, the effect of organotin on the surface treatment performance of siloxane was studied for application in cementitious materials, including surface hardness and waterproofing properties. Organotin had little effect on the waterproofing of cement paste treated with siloxane. However, the addition of organotin positively impacted the surface hardness of cement paste treated with MTMS, which increased by 105.1%. The function of organotin was analyzed through XRD, FTIR, SEM, and pore structure characterizations. Organotin can speed up the gelation of siloxane, thus consuming more portlandite in cement. The negative effect of the alkyl group could be partially reduced by promoting the condensation of the alkoxy group. This indicated that treating siloxane with organotin is valuable for improving the durability of cement-based materials, increasing their surface hardness without affecting waterproofing.

## 1. Introduction

The surface treatment of cementitious materials has received widespread attention from both the engineering field and academia [1]. Research on surface treatment focuses on protective sealer and coating systems, mainly including silicate solutions and polymer coatings [2,3,4]. Siloxane is a material with potential for surface treatment as the precursor of common sol–gel reactions, possessing abilities to react with cementitious materials and form a coating, which are the advantages of silicates and polymers, respectively. However, the development of the siloxane materials applied on cementitious materials is limited despite being the earliest protective material used in construction engineering [5,6,7,8,9,10]. For siloxane to achieve good surface treatment performance, the contained functional groups are an essential factor, as alkyl groups provide hydrophobicity, while alkoxyl groups have reaction capability. Therefore, further improving the realization and employment of functional groups is important to develop better siloxane-based materials to be applied in the surface treatment of cementitious materials.

The effects on the surface hardness and impermeability of cementitious materials determine the surface treatment performance, influencing the durability of construction [11,12]. The mechanism of silicate solution is to generate calcium silicate hydrate (CSH) gels in situ, filling pores in the cementitious matrix by reacting with portlandite [13,14,15,16,17,18,19,20,21,22]. The increase in surface hardness and impermeability achieved using silicate solution depends on the amount of CSH gels generated and the filling results. The coating covering the cementitious matrix is the protection mechanism of polymer coatings [23,24,25,26,27,28]. The properties of the coating determine the protective performance, benefiting from the hydrophobicity of the polymer, and are limited by the durability of the polymer. Siloxane, whether used for surface treatment or directly added to cement’s hydration process, can make the surfaces of cementitious materials hydrophobic. The working mechanism of siloxane is reported to be the formation of a coating through a bonding interaction [29,30,31,32,33]. This mechanism can explain the contribution of siloxane to the improvement in surface hydrophobicity; however, it cannot reflect its effect on surface hardness, and reports of the effect of siloxane on surface hardness are lacking. In addition, the hydrophobic properties of such surfaces formed by films are susceptible to mechanical damage. Therefore, it is essential to focus on the mechanical properties of the surface while determining the effects of siloxanes on the hydrophobicity of cementitious materials.

Considering the reaction occurring in the surface treatment of siloxane, the capability for reaction is important. The chemical environment of cementitious materials cannot allow for the complete condensation of siloxanes in a short surface treatment time. Therefore, adapting catalysts to accelerate the condensation process of siloxanes would be a good idea, as this could improve the early performance of the surface treatment. Catalysts commonly used for the condensation of siloxanes are acids and bases, which need heat for processing. In addition, acids and bases can have harmful effects on cementitious materials. Therefore, using organotin might be a better option to improve the performance of siloxane, as it has been reported to be used in producing silicone elastomers and coatings [34,35,36,37]. Organotin is considered both a catalyst and a crosslinker for siloxane, thereby enhancing the condensation degree of siloxane in the gelation process. The reaction equations between organotin and siloxanes are as follows:

(1) $${Bu}_{2}Sn{\left(OCOR^{\prime }\right)}_{2}+H_{2}O\to {Bu}_{2}Sn\left(OCOR^{\prime }\right)\left(OH\right)+HOCOR^{\prime }$$

(2) $${Bu}_{2}Sn\left(OCOR^{\prime }\right)\left(OH\right)+Si{\left(OR\right)}_{n}{R^{\prime \prime }}_{4-n}\to {Bu}_{2}Sn\left(OCOR^{\prime }\right)OSi{\left(OR\right)}_{n-1}{R^{\prime \prime }}_{3-n}+ROH$$

(3) $$Si{\left(OR\right)}_{n}{R^{\prime \prime }}_{4-n}+H_{2}O\to Si{\left(OR\right)}_{n-1}{R^{\prime \prime }}_{3-n}(OH)+ROH$$

(4) $${Bu}_{2}Sn\left(OCOR^{\prime }\right)OSi{\left(OR\right)}_{n-1}{R^{\prime \prime }}_{3-n}+Si{\left(OR\right)}_{n-1}{R^{\prime \prime }}_{3-n}\left(OH\right)\to \;{\left(OR\right)}_{n-1}{R^{\prime \prime }}_{3-n}SiOSi{\left(OR\right)}_{n-1}{R^{\prime \prime }}_{3-n}+{Bu}_{2}Sn\left(OCOR^{\prime }\right)\left(OH\right)$$

In these reactions, *R*, *R*′, and *R*″ are the alkyl groups, *n* = 2, 3, 4. In the surface treatment of cementitious materials, the chemical environment of the siloxane reaction is complex, and the effect of organotin on the surface treatment performance of siloxane has not yet been reported.

The goal of this study was to apply organotin to siloxane to carry out the surface treatment of cementitious materials. To better study the effect of organotin catalysis on different siloxanes, siloxanes containing different alkoxyl groups in different quantities were chosen as the objects of this work. We studied the effects of siloxane and siloxane with organotin on the surface hardness and waterproofing performance of cementitious materials. Although concrete is more reflective of real application conditions, surface treatment with siloxanes has greater effects on cement, and the presence of aggregates may affect the results of the hardness tests. Therefore, we conducted this study on cement paste. Besides cement paste, cement hydration particles were pressed into pieces to study the surface hardening mechanism of siloxane. The waterproofing performance was studied according to the water absorption and surface hydrophobicity. The form and structure of the siloxane surface treatment on cementitious materials were morphologically analyzed, and the mechanism of the siloxane surface treatment and the effect of organotin were analyzed according to the compositional and structural changes in the cementitious materials.

## 2. Experimental Section

### 2.1. Materials

The cement used in this study was obtained from the China Building Materials Academy (Beijing, China). Tetramethoxysilane (C_4_H_12_O_4_Si, TMOS), tetraethyl orthosilicate (C_8_H_20_O_4_Si, TEOS), trimethoxymethylsilane (C_4_H_12_O_3_Si, MTMS), and dimethoxydimethylsilane (C_4_H_12_O_2_Si, DMDMS) bought from Aladdin Ltd. (Shanghai, China) were selected as siloxane as they have different alkoxyl groups and varying amounts of alkyl groups. The organotin catalyst selected for this study was dibutyltin diacetate, purchased from Aladdin Ltd. (Shanghai, China).

### 2.2. Preparation of Cement Paste and Surface Treatment

The cement paste was prepared using a water/cement ratio of 0.5 in a mold with dimensions of 40 × 40 × 10 mm^3^ at a temperature of 20 °C and a humidity of 50%. The solidification time was 24 h. Cement pastes were cured at a temperature of 20 °C and a humidity of 95% for 28 days after demolding. The curing condition ensured that cement hydration was complete. The surface of the cement paste in contact with the bottom of the mold was used for surface treatment. Each siloxane needed to process six cement pastes at an amount of 1 mL, half of which contained 0.001 mL of organotin. The minimum organotin amount was used to ensure that the silicone would achieve gelation in a limited surface treatment time. We ensured that the surfaces of the treated cement pastes were completely covered by siloxane.

### 2.3. Preparation of Cement Hydration Piece and Surface Treatment

The cement hydration particles were obtained by crushing and grinding the obtained cement paste into powders that could pass through a 60-mesh sieve, yielding powders smaller than 300 μm. Then, 0.5 g of cement hydration particles was placed in a mold and pressed into a circular piece with a diameter of 13 mm under a pressure of 10 MPa for 60 s. The thickness of the obtained pieces was about 2 mm.

The piece was placed in a container with a diameter of 30 mm and immersed in 3 mL of siloxane for 24 h. The depth of liquid was over 4 mm, ensuring complete immersion of the piece. This surface treatment method allowed the surface hydration particles to be in contact with enough siloxane. After an immersing period of 24 h, the cement pieces were taken out. The sample amount of the cement hydration pieces was the same as that of the cement paste sample.

### 2.4. Hardness Test

The surface hardness of both the cement pastes and the cement hydration piece was tested using a micro-hardness tester (HVS1000, Shanghai, China). The load was 0.98 N with a holding time of 15 s. For each sample, 20 points were tested to ensure data reliability. The average and standard deviations were obtained to represent the final value of surface hardness.

### 2.5. Water Absorption and Contact Angle Test

Water absorption and contact angle tests were carried out on the cement paste samples. The samples were dried under 70 °C and then placed in deionized water, ensuring the samples were completely wet. The masses of cement paste were recorded at 0 h, 1 h, 2 h, 3 h, 6 h, 9 h, 12 h, 18 h, and 24 h. The mass change ratio was the water absorption rate. This test was repeated three times for data reliability. A drop of water was placed on the surface of the cement paste and photographed to observe the contact angle between the water droplets and the surface.

### 2.6. Characterization

The chemical structures of the sample surfaces were characterized by a Fourier transform infrared spectroscopy (FTIR, Nicolet iS5, Thermo Scientific, Waltham, MA, USA) instrument equipped with a universal attenuated total reflectance (ATR) sampling accessory. The scans were performed 32 times with a resolution of 4 cm^−1^. The crystal structures were analyzed using an X-ray diffraction spectrometer (XRD, Bruker D8 Advance, San Jose, CA, USA). The scan rate was set at 1°/min with a step size of 0.02°. The morphology was observed using field emission scanning electron microscopy (SEM, ZEISS Gemini SEM 360, Oberkochen, Germany). The elemental composition of the sample surfaces was determined using an onboard energy-dispersive X-ray spectrometer (EDS). The nanoporous structures of various samples were measured using a surface area and porosity analyzer (Micromeritics ASAP 2460, Norcross, GA, USA) based on the Brunauer–Emmett–Teller (BET) theory. The test specimens were cubes measuring 3 mm × 3 mm × 2 mm in size.

## 3. Results and Discussion

### 3.1. Waterproofing

**Figure 1:**
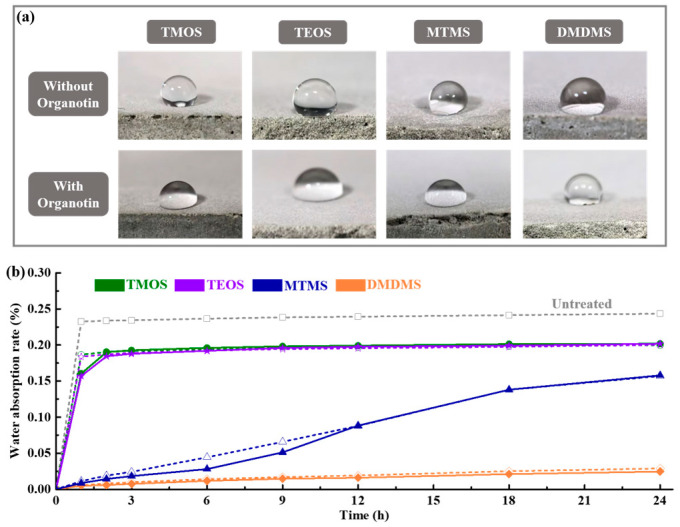
(**a**) Surface hydrophobic effect and (**b**) water absorption rate of cement paste treated with siloxanes. Dashed lines with hollow symbols refer to samples without organotin, and solid lines with solid symbols refer to samples with organotin.

Figure 1 shows the surface hydrophobicity and water absorption rates of each cement paste treated with various siloxanes with or without organotin. The addition of organotin decreased the surface hydrophobicity of the cement paste treated with TMOS, TEOS, and MTMS, while it had little effect on the cement paste treated with DMDMS, as shown in Figure 1a. Without organotin, the chemical environment of the cementitious materials did not allow the siloxane to form a hydrophilic film completely through a condensation reaction while retaining the alkoxyl groups. Organotin induced the further condensation of siloxanes in the cementitious materials, resulting in a change from a hydrophobic film formed by siloxanes to hydrophilic silica particles on the surfaces of the cementitious materials, leading to a decrease in hydrophobicity. However, the change in surface hydrophobicity had no obvious impact as the water absorption rates of the cement paste treated with and without organotin were almost the same at 24 h, as shown in Figure 1b. Surface treatments with TMOS and TEOS have a similar effect on the water absorption rate, and the addition of organotin finitely delayed the water absorption speed. Evidently, the cement paste treated with DMDMS presented the lowest 24 h water adsorption rate, showing it has the best waterproofing. Surface treatment with MTMS could significantly decrease the water absorption rate in the first six hours, and the addition of organotin also promoted a decrease. However, the water absorption rate increased faster with an increased water immersion duration. This indicates that an increased amount of alkyl groups has a great benefit for the waterproofing of cementitious materials.

### 3.2. Surface Hardness

**Figure 2:**
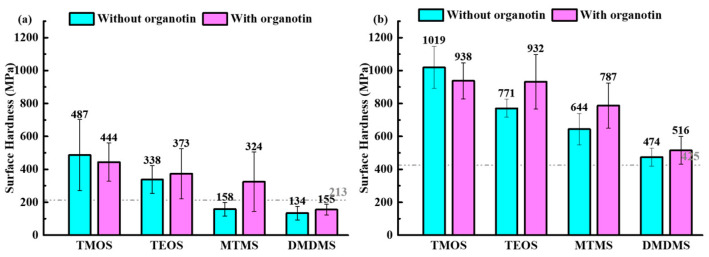
Surface hardness of (**a**) cement paste and (**b**) cement hydration piece treated with siloxanes. The gray dashed line represents the hardness value of the untreated sample.

The surface hardness of the cement paste treated with siloxanes is presented in Figure 2a,b shows the surface hardness of the cement hydration piece which could ignore the influence of micropores on cement hardness. The order of surface hardness enhancement from high to low was as follows regardless of whether organotin was used: TMOS > TEOS > MTMS > DMDMS, shown in Figure 2. According to the surface hardness change in the cement hydration pieces, it could be inferred that the surface hardness of the cement paste was increased by increasing the hardness of cement hydration, as they had a similar enhancement rule. We determined that the alkoxyl groups of the siloxane had a positive effect on hardness, while the sample of DMDMS had a negative surface hardness that was even lower than that of the untreated cement paste. The effect of organotin on hardness was different for various siloxanes. The increasing surface hardness rates of the cement paste that underwent surface treatment with organotin relative to that without organotin were −8.8%, 10.4%, 105.1%, and 15.7% for TMOS, TEOS, MTMS, and DMDMS, respectively. This indicates that organotin enhanced the hardness performance of siloxane by affecting the alkoxyl group. Dialkoxysiloxane limited the function of organotin as only a liner structure could be formed. However, the decreased hardness after surface treatment with TMOS and organotin might be due to excessive gelation by the function of organotin. Hence, using siloxane and a suitable amount of organotin would be essential for improving the surface hardness of cementitious materials.

### 3.3. Chemical Composition

**Figure 3:**
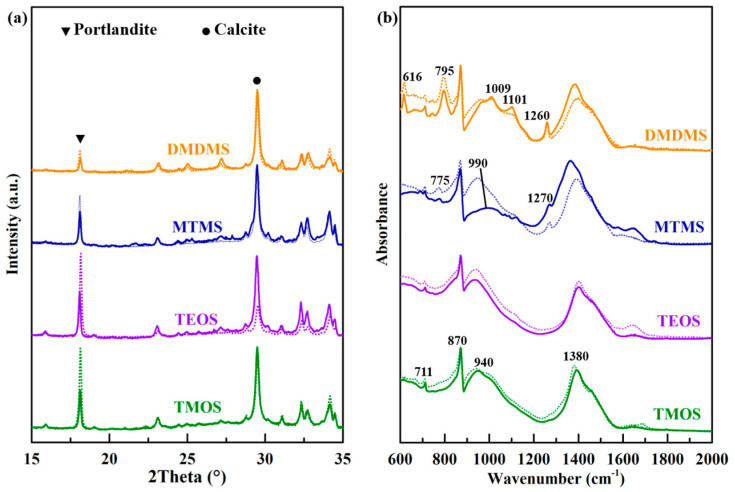
(**a**) The XRD patterns and (**b**) FTIR spectra of cement piece surfaces treated with siloxanes, with the dotted curve corresponding to siloxane without organotin and the solid curve corresponding to siloxane with organotin.

The effect of organotin on the chemical compositions of cementitious materials treated with siloxane was analyzed using the results of XRD shown in Figure 3a and the results of FTIR shown in Figure 3b. The diffraction peak intensity of portlandite decreased with the addition of organotin, as shown in the normalization curve of the XRD patterns. The FTIR spectra show the same chemical structure for surface treatment with TMOS, TEOS, and DMDMS with and without the addition of organotin, and the absorption band corresponding to the stretching vibration of Si-O-Si changed from 940 cm^−1^ to 990 cm^−1^ in the FTIR spectrum of the sample treated with MTMS and organotin. The peaks at 1383 cm^−1^, 870 cm^−1^, and 711 cm^−1^ point to the stretching vibration and deformation vibration of calcite. The alkyl group was introduced into cement hydration through treatment with MTMS and DMDMS due to the band at 1270 cm^−1^ and 1260 cm^−1^ corresponding to the vibration of the C-H bond, and the bands at 775 cm^−1^ and 795 cm^−1^ corresponding to the vibration of the Si-C bond. Broad adsorption bands at 940 cm^−1^ were assigned to CSH gels of cement. The addition of organotin had a greater effect on MTMS and DMDMS, as the bands at 990 cm^−1^, 1009 cm^−1^, and 1101 cm^−1^ represent the characteristic peaks of silica gels [38,39,40,41]. Through the chemical composition change with the addition of organotin, it could be inferred that organotin promoted the reaction between siloxanes and portlandite, and the gelation of MTMS and DMDMS, but it had little effect on the chemical structure of the production generated by TMOS and TEOS. In general, the increased condensation degree of siloxanes resulted in better surface hardening of the cementitious materials. However, TMOS was an exception, which may have been due to the fact that the rapid condensation reaction led to granulation rather than adhesion. In addition, the elevated condensation reaction also led to a decrease in the film-forming abilities of siloxanes, further consuming the hydrophobic alkoxyl groups.

### 3.4. Pore Structure

**Figure 4:**
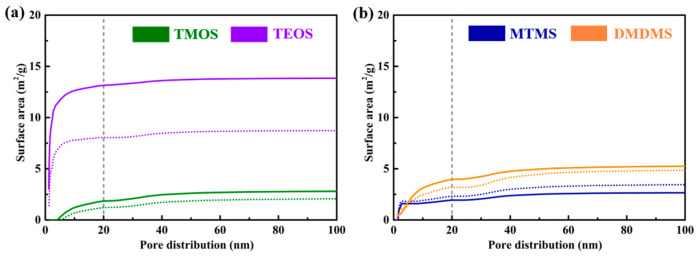
The surface areas of cement hydration pieces treated with (**a**) tetraalkoxysiloxane and (**b**) alkylakoxysiloxanes, with the dotted curve corresponding to siloxane without organotin and the solid curve corresponding to siloxane with organotin.

The surface area results obtained using the BET theory presented the nanoparticle distribution in cement treated with various siloxanes, as shown in Figure 4. For TMOS and TEOS, which is tetraalkoxysiloxane, the surface area contained pores smaller than 20 nm, as shown in Figure 4a, which was formed by gels in the treated cement. The addition of organotin increased the cumulative surface area to pores smaller than 20 nm, meaning the amount of gel increased. The difference between surface treatment with TMOS and TEOS is that the surface area of the generated gels of TEOS is larger than that of TMOS. For alkylakoxysiloxanes, the number of pores smaller than 20 nm also increased with the addition of organotin in the cement treated with MTMS, which is in contrast to the cement treated with DMDMS. In addition, the surface area containing pores ranging from 20 nm to 100 nm decreased with the addition of organotin. This indicated that the organotin was helpful in reducing the shrinkage occurring during the gelation of alkyl alkoxysilanes. The BET results show that the increase in nanoproducts in the siloxane surface treatment did not have a negative effect on the surface hardness of the cementitious materials, and it could improve waterproofing in a short period of time. The reduction in pores between 20 and 40 nm was beneficial to the increase in surface hardness.

### 3.5. Surface Layer Morphology

**Figure 5:**
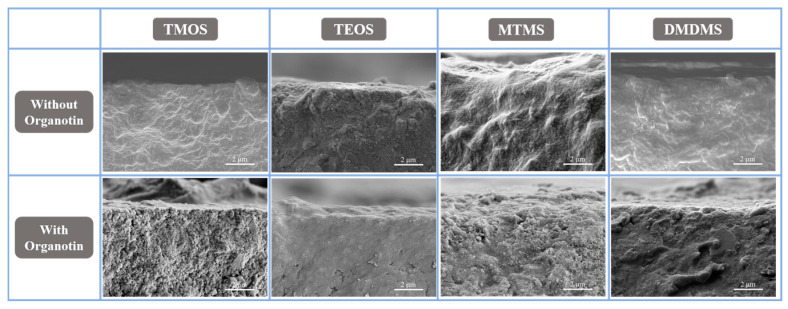
SEM images of cement hydration pieces treated with various siloxanes with and without organotin.

Scanning electron microscopy visually reflected the effect of the surface treatment on the cementitious materials, and the product generated by siloxane can be observed in Figure 5. Surface treatment with TMOS, MTMS, and DMDMS formed a coating on the surfaces of the cement hydration particles. The gelatinous coating further shrank towards the cement hydration particles with the addition of organotin. For TEOS, the function of organotin, in contrast, promoted the formation of siloxane coating. It can be inferred that the generation of the gelatinous coating was the result of an incomplete reaction, and the gel’s formation could be sped up by organotin. The gelatinous coating generated by siloxane was silica gel. And with the consumption of portlandite, the silica gel could transform into calcite silica gel through adsorbing Ca^2+^. The hardness of cement could be increased by a gelatinous coating without the alkyl group. The alkyl group weakened the stiff structure of the gelatinous coating as the stiff structure was formed by the condensation of siloxane by alkoxyl groups. But excessive condensation would break the bonding structure between colloidal particles. Therefore, controlling the gelation of siloxane is beneficial for surface treatment performance regarding the hardness of cementitious materials.

## 4. Conclusions

The function of organotin as a neutral catalyst of siloxane applied in the surface treatment of cementitious materials, as well as the surface treatment performance regarding waterproofing and surface hardness, were studied by comparing the surface treatment results of siloxane with and without organotin. While the addition of organotin had little effect on the waterproofing of cement treated with siloxane, it improved the hardening performance of siloxane in the cementitious materials, except for TMOS. The best improvement was achieved in the surface treatment with MTMS, as the surface hardness of the treated cement paste increased by 105.1% with the addition of organotin. The function of organotin is to speed up the gelation process of siloxane in cementitious materials, increasing the surface hardness of cement by forming a highly gelatinous coating on cement hydration particles. The formed gelatinous coating consisted of calcium silica gels, which are close to CSH gels and, thus, consumed portlandite, the hydration production of cement. However, excessive gelation of siloxane negatively affects the hardness enhancement, which was indicated in the performance of TMOS with organotin. Surface hardness enhancement is meaningful for the surface treatment of cementitious materials, as the control of gelation of siloxane is an essential factor in surface treatment performance.

Of course, long-term exposure to organotin compounds may be toxic to humans. While the functional organotin would be immobilized in the silicon–oxygen chain, further studies are still needed to determine whether toxic effects exist. In addition, this surface treatment method is mainly used for portlandite cement, and further studies should pay attention to the hardening effect of siloxane with organotin on other cements.

## Data Availability

The original contributions presented in this study are included in the article. Further inquiries can be directed to the corresponding author.
